# Motivations, understandings, and experiences of open‐access mega‐journal authors: Results of a large‐scale survey

**DOI:** 10.1002/asi.24154

**Published:** 2019-01-22

**Authors:** Simon Wakeling, Claire Creaser, Stephen Pinfield, Jenny Fry, Valérie Spezi, Peter Willett, Monica Paramita

**Affiliations:** ^1^ Information School University of Sheffield Regent Court, 211 Portobello, Sheffield, S1 4DP UK; ^2^ LISU, Centre for Information Management School of Business and Economics, Loughborough University Loughborough, LE11 3TU UK; ^3^ School of the Arts, English and Drama Loughborough University Loughborough, LE11 3TU UK

## Abstract

Open‐access mega‐journals (OAMJs) are characterized by their large scale, wide scope, open‐access (OA) business model, and “soundness‐only” peer review. The last of these controversially discounts the novelty, significance, and relevance of submitted articles and assesses only their “soundness.” This article reports the results of an international survey of authors (*n =* 11,883), comparing the responses of OAMJ authors with those of other OA and subscription journals, and drawing comparisons between different OAMJs. Strikingly, OAMJ authors showed a low understanding of soundness‐only peer review: two‐thirds believed OAMJs took into account novelty, significance, and relevance, although there were marked geographical variations. Author satisfaction with OAMJs, however, was high, with more than 80% of OAMJ authors saying they would publish again in the same journal, although there were variations by title, and levels were slightly lower than subscription journals (over 90%). Their reasons for choosing to publish in OAMJs included a wide variety of factors, not significantly different from reasons given by authors of other journals, with the most important including the quality of the journal and quality of peer review. About half of OAMJ articles had been submitted elsewhere before submission to the OAMJ with some evidence of a “cascade” of articles between journals from the same publisher.

## Introduction

Open‐access mega‐journals (OAMJs) have over the last decade proved to be an important and at times controversial innovation in scholarly communication. OAMJs combine four major characteristics that their publishers argue enable them to contribute in new ways to research publishing (Björk, 2015; Spezi et al., 2017). First, they are large scale: publishing larger volumes of articles compared with most conventional journals; the two largest, *PLoS One* and *Scientific Reports*, published 20,395 and 24,318 articles in 2017, respectively. Second, OAMJs tend to have a wide scope, often covering an entire disciplinary area or more; for example, *BMJ Open* covers all of medicine, *Royal Society Open Science* covers all disciplines across science, engineering, and mathematics. Third, mega‐journals publish all of their output in an open‐access (OA) form, typically supported by prepublication article‐processing charges (APCs). Finally, OAMJs deploy an approach to quality assessment that limits peer review to cover technical or scientific “soundness” only. Judgments on an article's novelty or significance or relevance to a particular readership (criteria important in reviews for conventional journals) are considered “subjective” and are not taken into account. For example:

The prepublication peer review process focuses on whether the manuscript is technically correct and original. Concepts of ‘timeliness,’ ‘significance,’ or ‘importance’ are evaluated by the community post‐publication through the implementation of online commenting and ranking tools. (*AIP Advances*)
PeerJ evaluates articles bbased only on an objective determination of scientific and methodological soundness, not on subjective determinations of ‘impact,’ ‘novelty’ or ‘interest..’ (*PeerJ*)

To add to these four primary features, Björk (2015) identified a set of secondary OAMJ features: a “moderate APC,” a “high‐prestige” publisher, use of large numbers of academic editors, provision of “reusable graphics and data,” use of altmetrics and commenting functionality, and a rapid publication process.

OAMJs have given rise to controversy and debate among researchers, publishers, and other stakeholders in scholarly communication ever since the first journal of this type, *PLoS One*, was launched in 2006, and particularly after 2011, when a number of other publishers set up “*PLoS One‐*like” titles (Spezi et al., 2017). Some commentators have characterized OAMJs as collections of lower quality content that has been rejected by journals of first resort, often enabled by publishers “cascading” articles from their titles with higher rejection rates. More recently, mega‐journals have been the subject of various scholarly studies, some of which arise from our own 2‐year research project investigating different aspects of the mega‐journal phenomenon. This mixed‐methods study comprised five main research phases: (a) a literature review, (b) bibliometric study, (c) interviews with publishers and editors, (d) focus groups with researchers, and (e) an international survey of authors. The phases were carried out sequentially to build up a multifaceted picture of OAMJs. This article reports the results of Phase 5 of the project: analyzing survey respondents' motivations for publishing in their chosen journal, their understandings of the main characteristics of that journal, and their experiences of the publishing process. It compares the perspectives of respondents who chose OAMJs with those who contributed to other types of journals, both OA and subscription titles. As such, it is the first publication to report a survey specifically comparing author perspectives for both OAMJs and other journals, and therefore provides new insight into OAMJs and their place in the scholarly communication landscape.

The article addresses the following research questions:

- RQ1. What are the major factors that motivate authors to publish in open‐access mega‐journals?
- RQ2. To what extent are authors satisfied with the experience of publishing in a mega‐journal and how does this relate to their future publishing intentions?
- RQ3. To what extent do authors understand the design of mega‐journals and how does this influence their behaviors?
- RQ4. To what extent are mega‐journals publication venues of first choice for authors and what is the evidence of articles being cascaded from other publications?
- RQ5. How do the motivations and experiences of authors publishing in mega‐journals compare with those of authors of articles published in other types of journals?

This article is able to address these questions in particular detail because of the unusually large‐scale data set gathered from our survey, comprising more than 11,000 responses. The analysis brings to bear our findings from earlier phases of the mega‐journals project, as well as interacting with other published literature.

## Literature Review

Several studies have charted the growth of OAMJs (Björk, 2015; Domnina, 2016; Spezi et al., 2017; Wakeling et al., 2016). OAMJ output has continued to rise since the first mega‐journal, *PLoS One,* was launched in 2006. *PLoS One* and Nature's *Scientific Reports* dominate the overall outputs but other smaller OAMJs have also grown over the period: including *AIP Advances* (from 396 in 2013 to 1,238 in 2016), *BMJ Open* (959 to 1,773), and *PeerJ* (229 to 1,284).

Studies show that OAMJ authors come from a wide range of countries and disciplines. With regard to countries, authors generally reflect the distribution of the peer‐reviewed literature as a whole (Wakeling et al., 2016), although some titles show high levels of input from authors affiliated with non‐Western institutions. For example, Chinese authors are disproportionately represented in mega‐journals compared with the overall literature in *Medicine* (42.2% of articles compared with 12.7% of all articles in *Scopus* in the same subject area), *AIP Advances* (40.6% compared with 27.7%), and *Scientific Reports* (39.2% compared with 30.0%; Wakeling et al., 2016). We posited this may be because of author sensitivity to the Journal Impact Factor (JIF), with some academic reward schemes prioritizing publication in high‐JIF titles, and awareness of publishers' brands and reputations. Disciplinary coverage of mega‐journals, even those with a very broad scope, also varies: *PLoS One* publishes disproportionately in the biosciences (95% of articles in 2015), whereas *Scientific Reports* has a more even distribution of subjects (Wakeling et al., 2016). Perhaps the most important conclusion from this work is that there is no such thing as a “typical” mega‐journal, with each title having its own unique combination of author characteristics, citation profile, and subject scope.

With their approach to peer review being perhaps the most controversial component of mega‐journals, this issue has given rise to considerable debate, often articulated as a concern that OAMJs represent a lowering of quality (Spezi et al., 2017). Surprisingly, Björk and Catani's (2016) quantitative study and our own qualitative one (Spezi et al., 2018) appear to be the only major empirical studies on this to date. Björk and Catani (2016) compared citations of a sample of OAMJs with a sample of selective journals in information science. Although they found little difference between the citation patterns of mega‐journals compared with the selective titles, the small scope of the study means it is of limited use in understanding the effect of soundness‐only peer review. Our study on peer review reported a set of 31 detailed, semistructured interviews with OAMJ publishers and academic editors (Spezi et al., 2018). We found that, although mega‐journals were developed with clear aspirations to pursue soundness‐only peer‐review, in reality, considerations of novelty, significance, and relevance were evident in decision‐making processes in OAMJs. Reviewers were reported to sometimes carry out peer review for OAMJs in much the same way as for conventional journals, and publishers themselves had introduced a criterion for acceptance of articles that took account of their “worthiness” for publication (inevitably involving a novelty/significance threshold). Both of these studies raise issues meriting further exploration.

Our interviews also provided data on the perceptions of publishers on the factors influencing authors' choices of journal (Wakeling et al., 2017). These included the brand and reputation of the publisher, JIF, rapid publication of results, and the ability to find “a home” for other kinds of low‐impact studies (including null results). These results find some support in the two studies published to date on the motivations of OAMJ authors based on survey data. Solomon (2014) surveyed 665 authors of articles in *BMJ Open*, *PeerJ*, *PLoS One,* and *SAGE Open* aiming to understand why authors had chosen to submit to an OAMJ. A quarter of respondents (25.7%) selected “quality of the journal” as the most important, with the “journal's OA status” (14.2%), “Impact Factor” (14.0%), and “speed of review and publication” (12.6%) the other commonly selected factors. Solomon (2014) also found that half of all articles (52.6%) published in mega‐journals by survey respondents had previously been rejected by other journals. A similar study by Sands (2014), this time surveying 401 *BMJ Open* authors, found that over half (59%) selected the journal's OA status as a reason for submitting, with the “BMJ brand” (50%), “speed of review” (37%), and “reputation of the journal” (34%) other notable factors. Interestingly, only 13% of authors stated that the JIF influenced their choice. Geographical differences may exist, however. Shin (2017) argues that South Korean—and also Chinese—authors, who are under pressure to publish in high‐JIF journals to meet criteria for tenure, research funding, and financial rewards, particularly in the STM field, tend to favor short review and publishing times and higher acceptance rates, characteristics commonly associated with OAMJs.

These OAMJ‐focused studies may be compared with the literature addressing author motivations for journal publishing more generally. Pepermans and Rousseau (2016) suggest that factors influencing an author's choice of journal fall into three categories: author characteristics (career stage, perceived value of the journal on a CV, past experience with the journal), journal characteristics (prestige, quality of peer reviews, APCs, readership), and other research characteristics (including ethical considerations, impact on practitioners, and negotiation with coauthors). A large number of studies have attempted to understand the relative importance of these factors to authors in a variety of disciplines (for example, Bröchner & Björk, 2008; Gibler & Ziobrowski, 2002; Pepermans & Rousseau, 2016), and cross‐disciplinary studies have also been conducted (for example, Housewright, Schonfeld, & Wulfson, 2013; Nariani & Fernandez, 2012; Tenopir et al., 2016). Although there is some slight variation across disciplines, notions of journal quality and prestige, including JIF, regularly emerge as the most important factors influencing submissions, with audience and readership also often found to be significant. There are obvious points of connection with OAMJ‐focused studies but also clearly a need for studies that explicitly compare author motivations across different journal types, OAMJs and others—something this present study was designed to do.

Many of these findings were echoed in our focus groups with researchers at six U.K. universities (Wakeling et al., 2018). Awareness of the mega‐journal model was generally very low, with life scientists the most likely to be familiar with it. Perhaps the most prominent finding from the focus groups was the extent to which notions of community were found to influence researcher behavior—something that, with their broad scope and large scale, OAMJ do not seem to support. The idea of soundness‐only peer review was also troublesome to many participants. Not only did many feel that the lack of a filter for significance or importance would lead to information overload, but researchers appeared particularly to value high‐quality peer reviews as a means of refining and improving their work. They believed that mega‐journals, with their focus only on soundness, were unlikely to generate this kind of constructive feedback.

## Method

### *Overview*

The survey was designed to compare author perceptions and behaviors for OAMJs with four other journal types. This was to enable us to address a number of key issues identified in the earlier phases of our research and to compare with other previously published results, particularly those of Solomon's (2014) survey of OAMJ authors. The five journal types were:

1. OAMJ—broad subject scope, large size, open access, soundness‐only peer review policies.
2. Broad scope open access—similar to OAMJs with broad subject scope, large size, and OA, but with conventional peer review policies.
3. Open access—typically more focused subject scope with conventional peer review policies and full open access to articles. For this study, journals listed in the Directory of Open Access Journals (DOAJ) were considered OA.
4. Broad scope subscription—similar to OAMJs in broad scope and size, but with conventional peer review policies and a subscription model. *RSC Advances*, which was second only to *PLoS One* in terms of 2015 article output, was the only title included in this category.^1^
5. Subscription—generally narrower subject scope, conventional peer review policies, and subscription‐based publishing model (although individual articles may be OA on payment of a fee, the journal as a whole is not). Journals not included in the DOAJ were assumed to be subscription titles.

### *Questionnaire Development*

A questionnaire was developed based on the early findings of the qualitative stages of the research and drawing on the literature (Fry, Spezi, Probets, & Creaser, 2016; Solomon, 2014) for the wording of some questions. Five main areas were explored:

- Factors that influenced an author's choice of journal.
- Perceptions of aspects of the submission and publication process.
- Awareness of the peer review criteria used to assess the article.
- Whether the article had previously been submitted elsewhere, and, if so, whether resubmission had been at the suggestion of an editor or publisher.
- The likelihood of the author submitting another article to the same journal.

Throughout the survey, particular emphasis was placed on the fact that questions related to a specific article, which was clearly identified in the invitation email. The survey was piloted with around 10 researchers known to the project team, across various disciplines, and some changes made in response to their comments. A copy of the questionnaire is included at Appendix 1, with the differences (relating to questions about OA) between the versions distributed to authors for the different journal types marked.

### *Sampling Strategy*

The sampling methodology used was based on a stratified cluster sampling process. The selection of mega‐journals was based on earlier research conducted by the project (Wakeling et al., 2016), which identified 11 OAMJs that met Bjork's (2015) criteria, and were also indexed in *Scopus*. These 11 were augmented with an additional four mega‐journals that had been added to *Scopus* in the period since that earlier research. Appendix 2 shows these 15 OAMJs, along with some relevant bibliometric details.

Journals were considered OA if *Scopus* listed them as being registered on DOAJ. Source Normalized Impact per Article (SNIP), which is a field‐weighted measure of journal citation rates, was used to identify journals of comparable impact to OAMJs. The selection of subscription and OA comparison journals was therefore based on the following steps, using data obtained from *Scopus* and *Web of Science* (WoS).

1. A list was compiled of journals with a single high‐level subject area and 2015 SNIP in *Scopus* that were also indexed in *WoS*, and data on the numbers of articles published in 2015 extracted.
2. OAMJs, trade journals, and book series were excluded, to give a total 10,879 journals with 2015 SNIP and nonzero 2015 citable outputs data.
3. The mean and associated standard error of the SNIP was calculated for this set of journals, and journals with SNIP values within 3 standard errors of the SNIP for any of *PLoS One*, *SAGE Open*, *Scientific Reports*, *F1000 Research,* and *AIP* Advances (these OAMJ titles being considered representative of mega‐journal size and scope) were identified (1,589 titles).
4. The resulting list was sorted by 2015 article output.

The largest subscription and OA titles were selected for each high‐level subject area, regardless of the OAMJ with which their SNIP matched, or their more detailed subject areas. Titles that appeared not to be English language titles were excluded from consideration.

After some trial and error, we determined that email addresses were easiest to obtain from the PubMed database. To maximize the number of authors we could invite to take the survey, author email addresses were collected using a bespoke automatic harvesting script. This extracted all available email addresses for articles published in the selected journals in 2015 and 2016. In the case of 18 of the 47 journals in the sample, corresponding email addresses were not available on PubMed; in these instances we attempted to apply a modified script to the specific journal websites. This was successful in nine cases. For the remaining nine titles a pragmatic solution was adopted of replacing the title with the next largest in that discipline area. At the same time, the potential list of journals was expanded by including titles with SNIP values within 3 standard errors of the SNIP for not just the initial five representative OAMJs (*PLoS One*, *SAGE Open*, *Scientific Reports*, *F1000 Research,* and *AIP* Advances), but any of the 13 OAMJ titles with a 2015 SNIP. This was done to ensure that the final sample would include large comparison journals for each subject area and journal type.

The selection of broad scope journals drew on our knowledge of the academic publishing market, along with a review of the top 50 journals by article output on *Scopus*. Four journals were selected: *Nature Communications*, *eLife*, *Science Advances*, and *RSC Advances*. All four journals operate a traditional peer‐review process (that is, novelty/originality, interest/relevance, and importance/significance are considered prepublication). Author email addresses for articles published in these four journals were obtained using the same method described above.

The mailing software used (MS Outlook) limited batch invitations to 10,000, with one such batch email allowed per day. Time and resource constraints meant that we were unable to commit to the multiple additional days that would have been required to send emails to all authors for the largest titles. Instead, therefore, titles with more than 10,000 email addresses had a systematic random sample drawn, of up to 10,000. Appendix 3 shows the full list of titles included in the survey (as well as those for which we were unable to scrape email addresses), together with data on the manuscript management system used by each journal, the number of author emails extracted, the number of invitation emails sent, and the number of responses.

### *Data Collection, Coding, and Analysis*

The online questionnaire was created using the BOS Software package.^2^ Authors were sent a personalized email invitation to complete the survey, identifying the journal and article title that had resulted in their inclusion in the sample, over a 2‐week period in March 2017; nonrespondents were sent reminders in April 2017, and the survey closed at the end of April 2017. A total of 11,883 responses were received, a response rate of 13.0%, although this varied widely between journals. Of these, 5,751 were from mega‐journal authors, 3,017 broad scope, 1,697 from OA, and 1,418 subscription.

No questions were compulsory, and some respondents chose not to answer some questions. Some processing work was done prior to analysis. Detail on the disciplinary scope of the article was collected in four broad disciplinary groupings: health sciences; life sciences; physical sciences and mathematics; and social science, humanities, and arts. Where respondents ticked “other” and gave details, these were checked and in some cases allocated to a different broad group. Cases where disciplines from two or more broad subject groups were ticked were assigned the category “Interdisciplinary.”

The analysis was carried out using the IBM SPSS Statistics v. 22 analysis software (Armonk, NY). Confidence intervals are shown at a 95% confidence level. The fully processed data set is available in Figshare (https://dx.doi.org/10.17028/rd.lboro.7211924).

### *Characteristics of Respondents*

**Figure 1:**
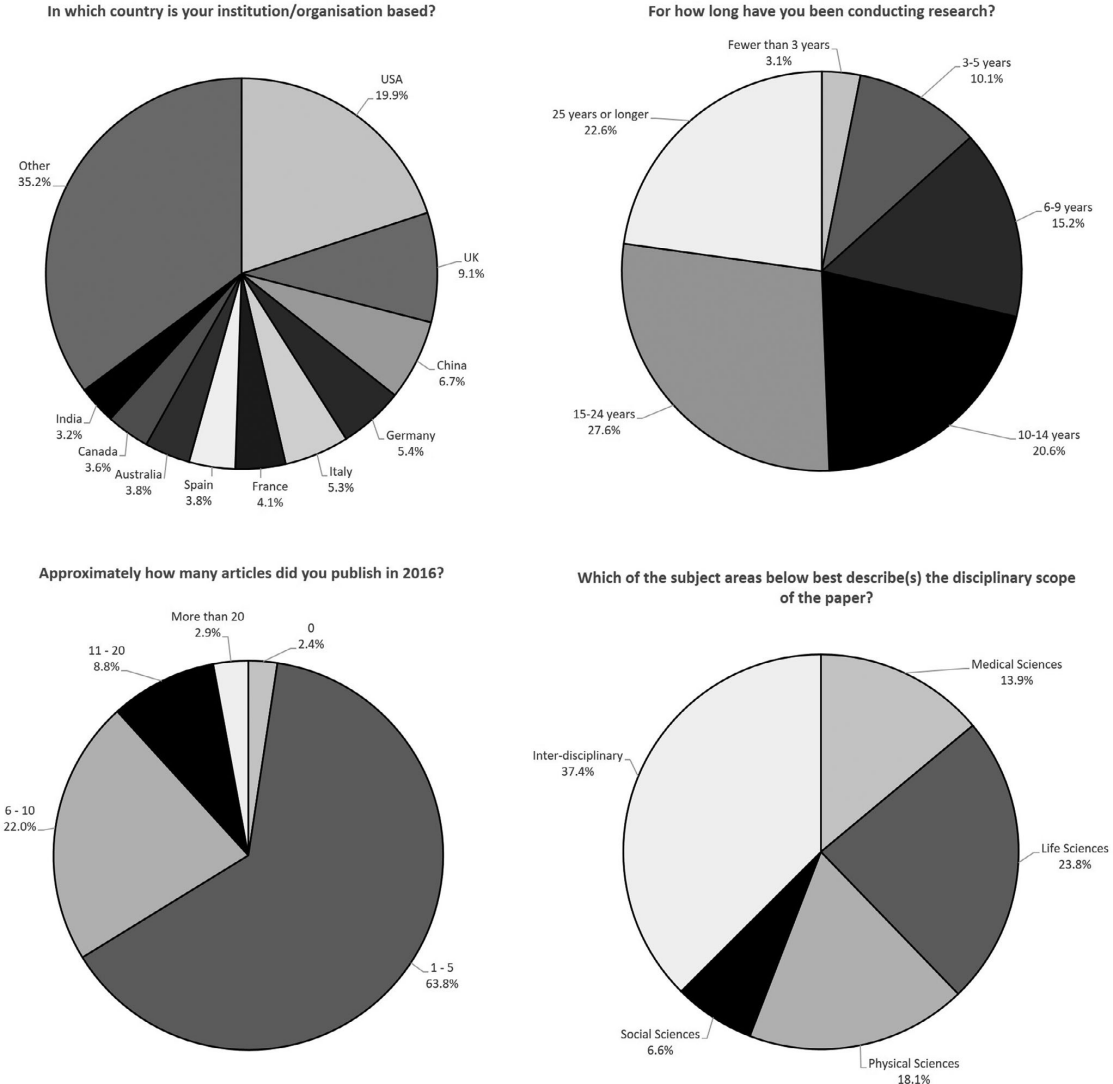
Characteristics of respondents.

Figure 1 shows key background data provided by respondents, the majority of whom (94.0%) were affiliated with universities or other academic institutions. In all, 132 countries were represented in the sample, although more than half of these (69) had fewer than 10 respondents. Over half of respondents (53.2%) had 15 or more years of experience conducting research. Of the total respondents, 13.3% could be described as early career researchers, with less than 5 years' experience, and around two thirds (63.8%) had published between one and five articles in any scholarly journal in 2016. More than one third (37.5%) of the respondents described the disciplinary scope of their article as relating to two or more of the broad subject groupings, and were categorized interdisciplinary. Authors of life science articles were most prominent, followed by physical sciences, health sciences, and humanities and social sciences.

## Results

### *Choice of Journal*

**Figure 2:**
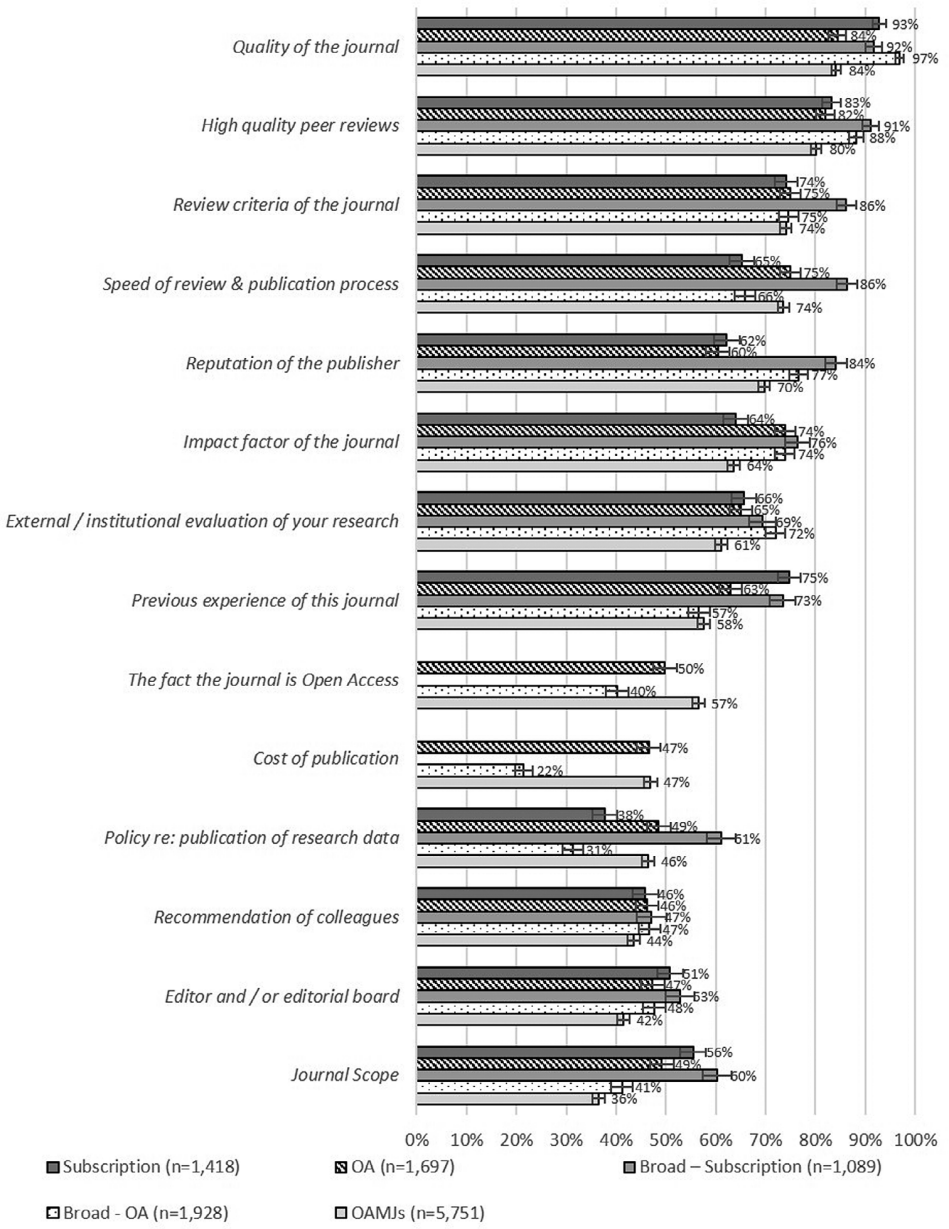
Proportion of respondents selecting “very important” or “extremely important” for each factor.

Respondents were asked to rate the importance of 14 factors in their decision to submit to a specific journal using a five‐point scale (1 = *not at all important*, 5 = *extremely important*). Figure 2 shows the proportion of respondents for each journal type who selected “very” or “extremely” important for each factor. Note that for “Journal scope,” respondents were first asked to state how broad or narrow they felt the scope of the journal was, then to rate the importance of this in their choice. It is striking that the importance attributed to the various factors is relatively consistent across the various journal types. Kendall's coefficient of concordance was used to calculate the degree of agreement between respondents for the different journal types, with the results showing significant and strong agreement in the ranking of the various factors (*W* = .886, *p* < .001).

Across all mega‐journals *Quality of the Journal* and *High quality peer reviews* emerged as the two most important factors. Given the four key characteristics of the mega‐journal model noted above, results for several of the other factors merit comment. *Journal Scope* did not emerge as a key driver of submissions to OAMJs, whereas *Review criteria of the journal* was considered “very” or “extremely” important by almost three quarters (74.1%; ±1.1%) of mega‐journal authors. However, this latter figure must be considered in the context of results for other journal types, all of which show a similar or greater proportion of “very” or “extremely” important responses. Relatively few authors appear to have considered the OA status of mega‐journals as important in their decision, although the proportion of OAMJ respondents who did say this was very or extremely important (56.5%; ±1.3%) is higher than for authors of articles published in OA and Broad‐OA journals (44.7%; ±1.6%).

A test of Kendall's coefficient of concordance for the relative importance placed on factors for author groups by mega‐journal title again showed relatively high levels of agreement (*W* = .747, *p* < .001). There was, however, notable variation between OAMJ titles for some factors (see Appendix 4 for a full breakdown of results by mega‐journal). For example, a large proportion of *PeerJ* authors (74.6%; ±3.9%) placed high value on the journal's OA status. *PeerJ* also had the highest proportion of very or extremely important responses for the *Cost of Publication* factor, perhaps because of the journal's innovative use of both institutional and individual membership models (69.6%; ±4.1%). It is notable that the other journal with high scores for this factor—*Royal Society Open Science* (66.9%; ±7.6%)—does not currently levy an APC at all.

There was also variation in the importance placed on JIF by OAMJ respondents. Authors of articles published in *Scientific Reports* (78.1%; ±2.2%) and *Medicine* (83.0%; ±4.9%) were the most likely to have viewed JIF as an important factor in their choice of driver, and it is perhaps unsurprising that these two journals had the highest JIFs of all mega‐journals for the years covered by the survey. Authors of articles published in journals without a JIF were unsurprisingly much less likely to have viewed JIF as an important factor.

A final point to note regarding these results is that most respondents identified numerous factors as being “very” or “extremely” important. Across all respondents, 71.3% rated 7 or more of the 14 factors this way, and 36.0% 10 or more. This supports earlier findings in the literature that authors are evaluating potential journals against a range of criteria, and that decisions on publication venues require a balancing of these factors.

OAMJ authors' responses to questions regarding factors influencing journal choice were also analyzed by discipline. Although chi‐square tests (excluding interdisciplinary responses) showed statistically significant differences between disciplines (*p* < .001) for all factors, this is to be expected given the large sample size of the survey. However, Cramer's *V* for all factors was found to be <.150, indicating very weak effect sizes.

Analysis of choice of journal factors by country revealed that of the 16 countries with more than 150 responses, Chinese, Taiwanese, and Spanish mega‐journal authors were the most likely to value the JIF of the journal, with 78.5% (±3.5%) of respondents from these countries rating JIF as “very” or “extremely” important, compared with 60.7% (±1.3%) of mega‐journal authors from other countries. American and British authors were the least likely to consider the journal's JIF. U.K. mega‐journal authors were much more likely to view the OA status of the journal as a “very” or “extremely” important factor in their choice of journal (74.8%; ±3.5%) than authors from any other country (54.5%; ±1.4%), perhaps reflecting U.K. funder requirements for the OA dissemination of results. Although these funder requirements do allow for Green OA dissemination of research outputs (that is, deposit in an institutional repository or similar), during the period covered by this research most of them (the publicly‐funded research councils and medical research charities, such as Wellcome) expressed a preference for Gold OA and also funded the payment of APCs (Jubb et al., 2017). Chinese mega‐journal authors were the least likely to value OA (32.4%; ±5.8%). Indian authors also produced interesting results, with many respondents (91.7%; ±4.3%) rating *Reputation of the Publisher* as “very” or “extremely” important. Indian respondents were also the most likely to consider the speed and cost of publication.

### *Perceptions of the Submission and Publication Process*

**Figure 3:**
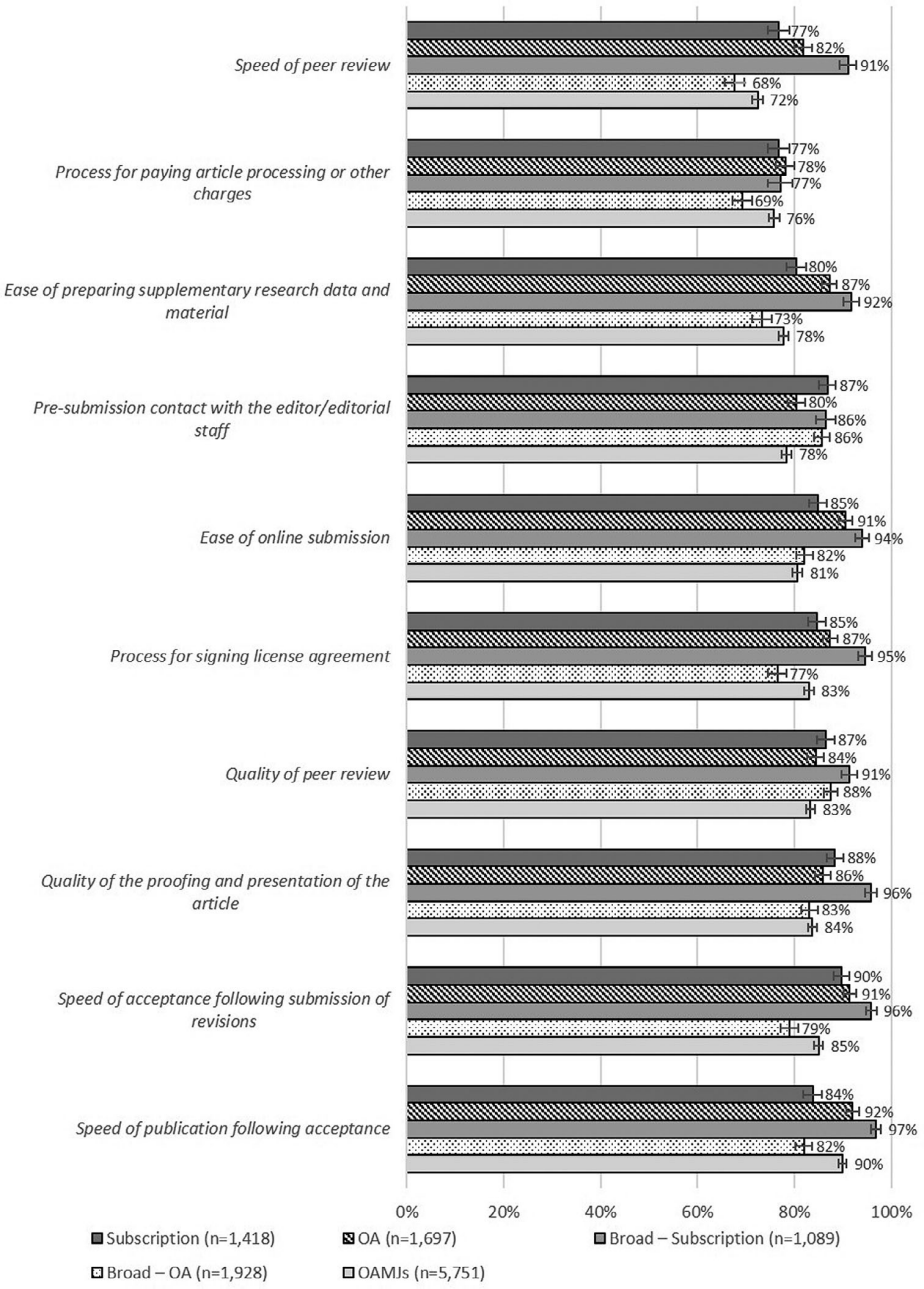
Proportion of respondents rating each aspect of the submission and publication process “good” or “excellent.”

Respondents rated 10 aspects of the submission and publication process on a five‐point scale (1 = *very poor*, 5 = *excellent*). Chi‐square tests revealed statistically significant differences between journal types (*p* < .001) for all aspects, again with relatively small effect sizes (Cramer's *V* < .100 in all cases). The results suggest that most authors were satisfied with most aspects, although in many cases the proportion of OAMJ authors who rated each aspect “good” or “excellent” was slightly lower than for other journal types (see Figure 3). Referring to the manuscript management systems used by the journals in the sample (see Appendix 3), it is interesting to note that a high proportion of OAMJs in our sample (46.7%) use in‐house systems, compared with OA (28.6%) and subscription journals (23.8%). Further analysis, however, revealed no clear relationship between manuscript management system and author satisfaction. This suggests that although manuscript management systems are no doubt relevant to authors' perceptions of journal publication processes, other factors are also at play.

It is also interesting to note that *Speed of peer review* was the aspect that OAMJ authors were least likely to rate as “good” or “excellent.” Examining responses by mega‐journal, we found that of the 15 OAMJs included in the sample, the two largest—*PLoS One* and *Scientific Reports*—ranked tenth or lower in each of the 10 aspects of the publication process. Both journals fared particularly poorly in *Speed of peer review*, with 13.4% (±1.9%) of *PLoS One* authors and 12.7% (±1.8%) of *Scientific Reports* authors rating this as “poor or “very poor.” It was also notable that *PLoS One*, which requires authors to publish raw research data alongside their article, received the lowest ratings for *Ease of preparing supplementary research data and material*, with only two thirds (66.8%; ±2.9%) of authors rating this process as “good” or “excellent.” In comparison, 85.1% (±2.2%) of authors for the four other mega‐journals that operate a similar mandate (*BMC Research Notes*, *F1000 Research*, *PeerJ,* and *Royal Society Open Science*) rated this aspect as “good” or “excellent.”

### *Awareness of Mega‐Journal Peer Review Policies*

The survey asked all respondents to indicate whether the journal in which their article was published considered each of five specific criteria during the peer review process:

1. Novelty/originality of the research.
2. Relevance/interest of the subject matter.
3. Importance/significance of the research.
4. Scientific/technical soundness of the research.
5. Clarity of argument and expression.

**Figure 4:**
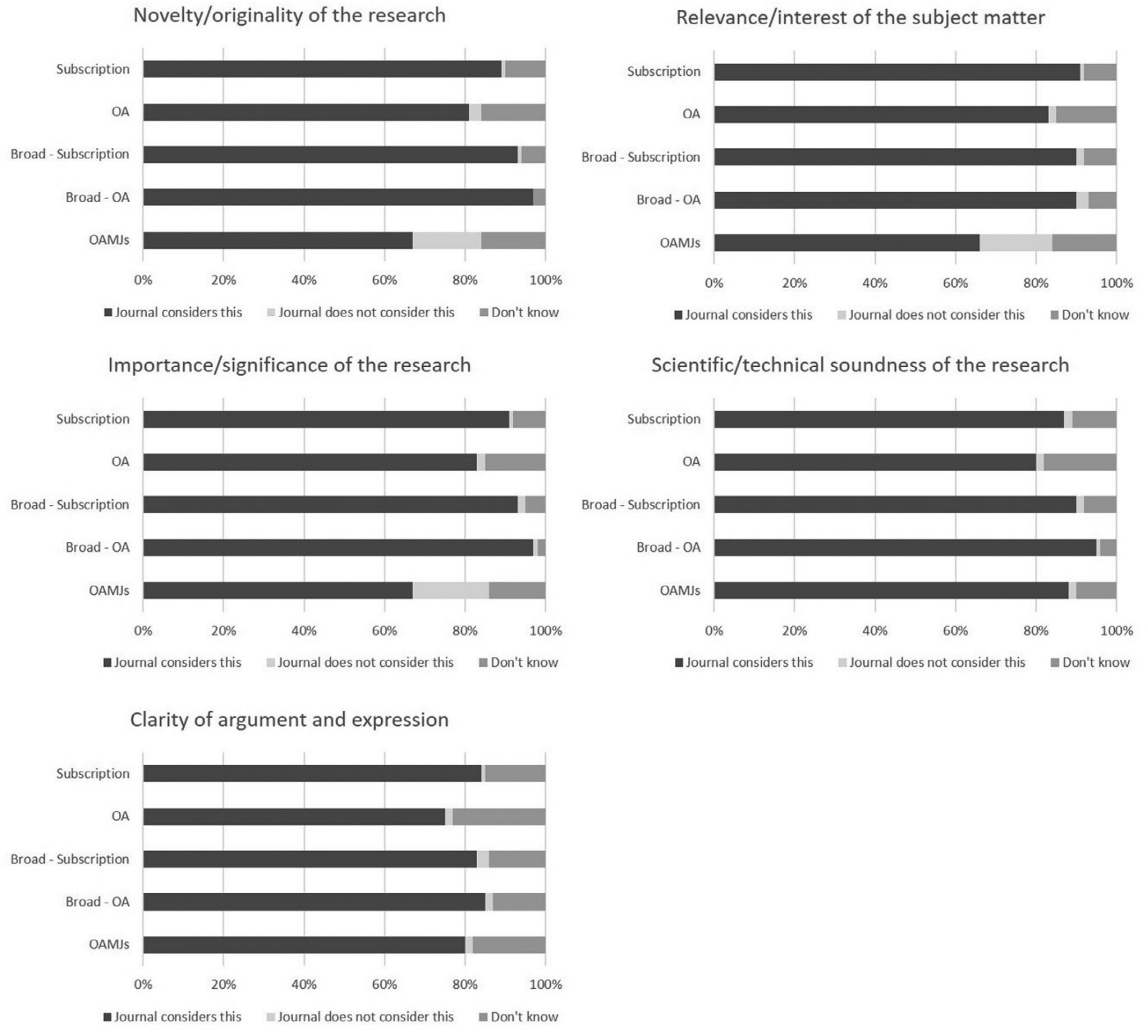
Responses to Q11: “When you submitted the article identified in the email invitation, were you aware which of the following peer review criteria were considered by this journal?” All confidence intervals are between ±0.2% and ±2.2%.

Given that the mega‐journal model ostensibly excludes all but scientific and technical soundness from the evaluation process, it was extremely surprising to find that a clear majority of all OAMJ authors surveyed believed on submission that their article would be reviewed for one or more of novelty, relevance, and significance. As Figure 4 shows, around two thirds of OAMJ respondents stated that their article had been reviewed against each of these criteria. Although there is some variation between OAMJ titles, it is clear that large numbers of authors are not aware that mega‐journals operate differently from traditional journals.

Perhaps most surprising are the findings relating to the evaluation of *Importance/Significance*. Although it is possible that respondents interpreted the terms *Novelty/Originality* and *Relevance/Interest* at their most basic level—that the journals determined that the work was not plagiarized, or completely beyond the scope of the journal—it is difficult to see how this could be the case for significance. Given how fundamental the notion of “soundness‐only” peer review is to the mega‐journal approach, it is noteworthy that for almost all mega‐journals, a majority of authors believed that the significance or importance of their work was to be considered prior to publication. In fact, only 18.5% (±1.0%) of mega‐journal authors said that they did not expect their work to be reviewed for significance, but to be reviewed for scientific soundness. It is also striking that of the 74.1% (±1.1%) of OAMJ respondents who said that review criteria had been very or extremely important in their decision about where to publish, two‐thirds (64.9%; ±1.5%) thought that the journal reviewed for significance.

Some OAMJs appear to have had more success in communicating their approach to quality control to authors. A chi‐squared test concerning whether the OAMJ was believed to review for significance revealed significant differences between OAMJ titles (*n =* 5,650; χ^2^ = 463.73, 28 df, *p* < .001), with a moderate effect size (Cramer's *V* = .203). *PeerJ* (44.5%; ±4.4%) had the fewest authors believing significance to be an assessment criterion, with figures for *Biology Open* (49.3%; ±11.6%) and *F1000 Research* (52.3%; ±7.9%) also low, although the relatively small sample size for these journals means the confidence intervals are high. In contrast, despite having operated a soundness‐only model for more than 10 years, and being undoubtedly the best‐known mega‐journal, 61.6% (±2.7%) of *PLoS One* authors appeared to be unaware of the journal's peer review model, a similar proportion to *Scientific Reports* (65.9%; ±2.5%). Authors publishing in *BMJ Open* (77.4%; ±3.2%) and *Medicine* (75.9%; ±5.6%) were the most likely to think that the journal would consider the significance of their article.

Analysis by country was also revealing. A chi‐square test revealed statistically significant differences between responses for OAMJ authors from the 16 countries with more than 150 responses (*n =* 3,830; χ^2^ = 141.48, 29 *df*, *p* < .001). Respondents from Taiwan (84.1%; ±7.9%), Brazil (81.5%; ±6.9%), India (79.5%; ±6.3%), and China (75.8%; ±5.3%) were the most likely to have believed significance was a criterion for publication, whereas authors from Germany (55.9%; ±6.1%), the United States (56.8%; ±3.3%), and the United Kingdom (58.3%; ±4.1%) were the least likely.

### *Article Resubmission and Cascade Rates*

**Figure 5:**
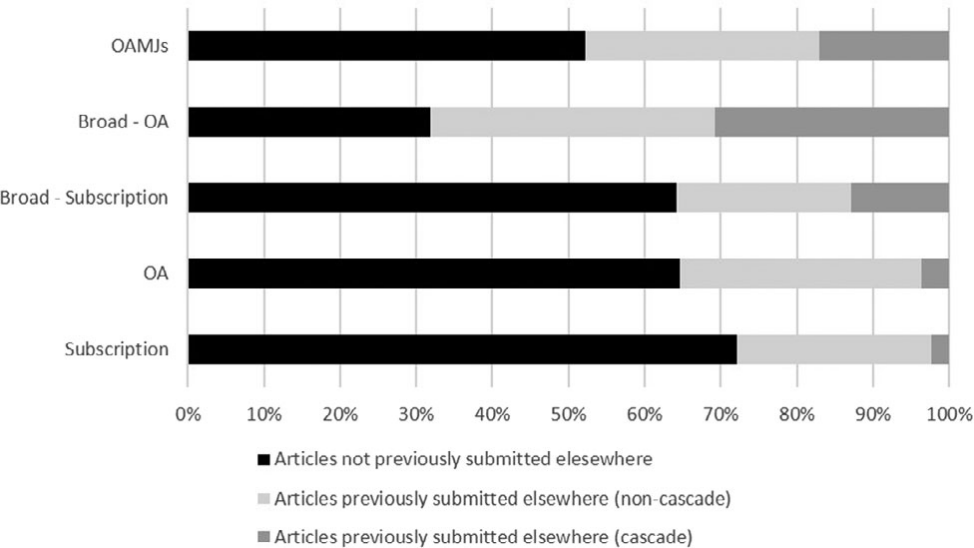
Proportion of authors who had previously submitted their article to another journal, and for whom resubmission to the eventual publishing journal was at the suggestion of an editor or publisher. Confidence intervals are between ±1% and ±3% for all journals.

An important goal of the survey was to better understand the proportion of mega‐journal articles that had previously been submitted to another journal, and whether the eventual submission to an OAMJ was on the suggestion of an editor or publisher from the original journal, that is, a cascade process. Figure 5 shows the results from the questions exploring this, and shows that across all OAMJs around half of articles (47.8%; ±1.3%) had previously been submitted to another journal. This rate is substantially higher than the equivalent figure for both OA (35.4%; ±2.3%) and subscription (27.8%; ±2.3%) journals. 10 of the 15 OAMJs have rates of previously submitted articles between 40% and 60%. *PeerJ* (38.4%; ±4.3%) and *F1000* (16.3%; ±5.7%) had the lowest rate of resubmissions, whereas around half of both *Scientific Reports* (52.6; ±2.6%) and *PLoS One* (43.9%; ±2.8%) authors said they had previously submitted their articles elsewhere.

Wide variation was found in the proportion of articles that were resubmitted at the suggestion of an editor or publisher. These figures indicate the extent to which publisher cascade policies are funneling articles rejected by other titles within the publisher's portfolio to a mega‐journal. Those mega‐journal publishers with few other titles (particularly *PeerJ* and *PLoS One*) understandably have relatively low cascade rates. Results for publishers with larger portfolios suggest considerable differences in the rates of cascade, with *BMC Research Notes* (28.3%; ±4.2%) and *Medicine* (30.8%; ±6.0%) showing higher proportions of articles coming from publisher suggested resubmissions than *BMJ Open* (17.5%; ±2.9%) and *SAGE Open* (9.9%, ±5.1%). *Scientific Reports* (18.9%; ±2.1%) was also found to have a relatively low cascade rate, a result which is of particular interest given previous suggestions that the journal's high JIF may be a consequence of articles originally submitted to more prestigious titles cascading to *Scientific Reports*. However, this figure should be viewed in the context of the large size of the journal: 18.9% of 2016 output represents more than 3,800 articles. The results for *Nature Communications* (included in the survey as a Broad OA journal) show that 44.6% (±2.8%) of articles had previously been submitted to another *Nature* journal, also suggesting that the publisher has implemented effective cascade practices for their more selective broad scope journal.

### *Likelihood of Submitting Another Article to the Journal*

**Figure 6:**
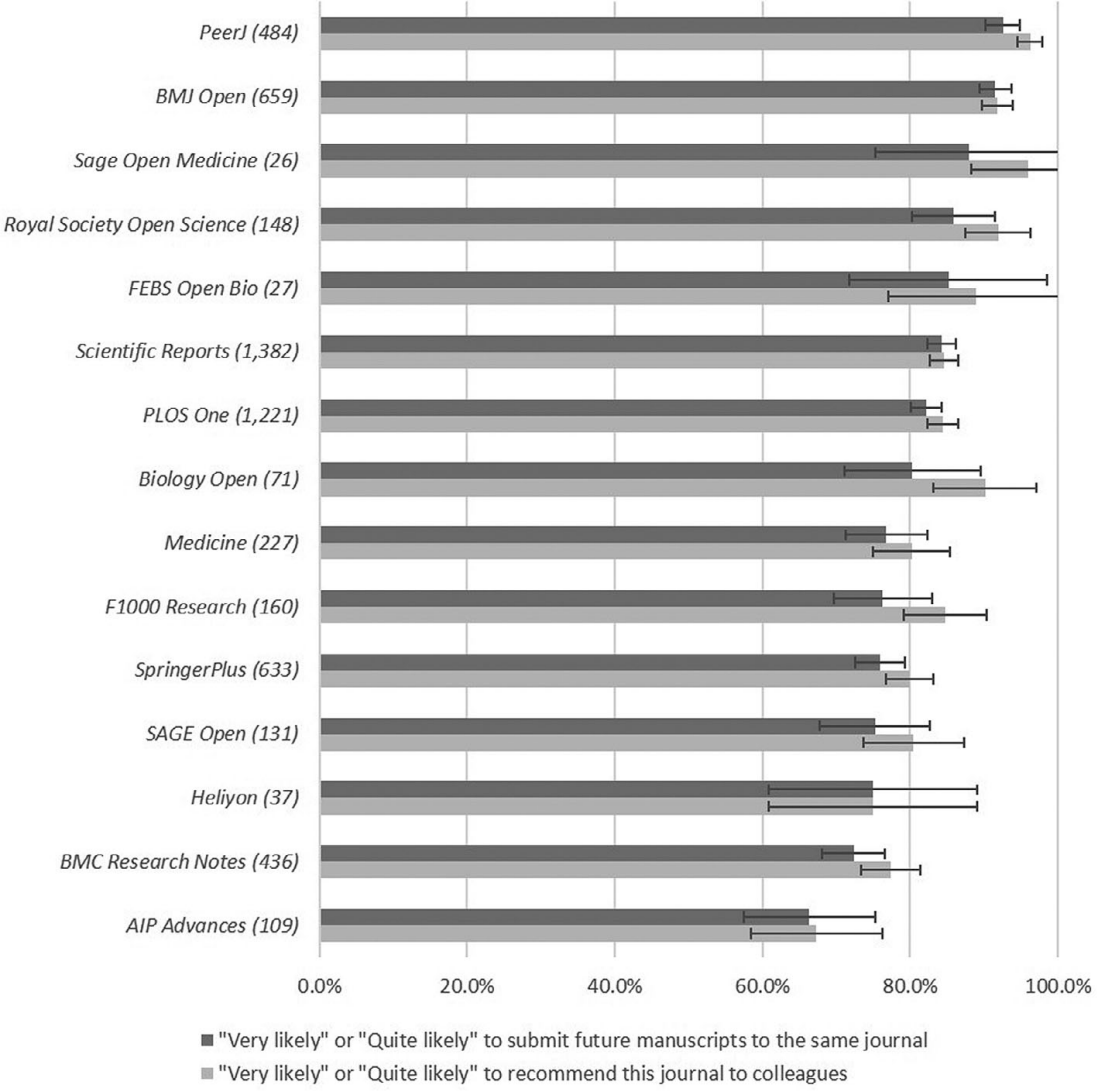
Proportion of authors “very likely” or “quite likely” to submit future manuscripts to the same journal, and to recommend the journal to colleagues. 95% confidence intervals shown.

The final questions asked participants how likely they were to submit another article to the same journal, and how likely they were to recommend the journal to colleagues (1 = *very likely*, 5 = *not at all likely*). The experience of most authors appears to have been positive, with 82.4% (±1.0%) of OAMJ authors saying they would be “quite” or “very” likely to publish another article in the journal. This is broadly comparable to the responses from authors of articles published in other types of journal, although the figure for subscription journals (93.4%; ±1.3%) is noticeably higher. As Figure 6 shows, *PeerJ* (92.5%; ±2.4%) and *BMJ Open* (91.5%; ±2.1%) achieved the highest positive results. Despite receiving marginally worse ratings for aspects of the submission and publication process, both *Scientific Reports* and *PLoS One* have high proportions of authors saying they would be likely to submit again (84.3%; ±1.9% and 82.2%; ±2.2%, respectively). For all 15 OAMJs, and for all types of journal, a slightly higher proportion of respondents said they were “quite” or “very” likely to recommend the journal to colleagues than said they were likely to submit again themselves!

## Discussion

One of the most noticeable features of our findings is that there was generally little difference between responses given for OAMJs compared with other journal types. These results provide clear answers to RQ1 and 5, regarding author motivation for publishing in OAMJs compared with other journals. Authors of articles in all journal types prioritize publishing their articles in high‐quality journals that facilitate high‐quality peer review. They want their work to be published speedily and efficiently. They are concerned about reaching intended audiences. They have a wide range of related factors to do with the quality of the journal and its production processes that they regard as important. These factors vary little across the different journal types covered in this study, as do apparent satisfaction levels arising from experience of publishing in different types of journals (RQ2 and 5). The fact that mega‐journals are OA often mattered less to our respondents than these other factors, although there is some geographical variation, with more U.K. authors citing OA to be important than others, probably reflecting the robust OA funder mandates in the United Kingdom (Johnson, Fosci, Chiarelli, Pinfield, & Jubb, 2017). Although OAMJs are sometimes discussed as potentially disruptive influences on the journal market, it is clear that they are dealing with authors who often have relatively conservative approaches to publishing.

The quality of the journal was most often cited as a factor influencing the decision to submit to the journal by authors in OAMJs and in the other journal types. This corroborates Solomon's (2014) findings where it was also the most important factor identified by his survey of OAMJ authors. It also resonates with well‐understood characteristics of the academic publishing market as a “reputation economy” in which authors seek to gain prestige from publishing in highly‐regarded journals (Fyfe et al., 2017). Perceptions of journal quality may reasonably be assumed to be closely linked with the reputation of the publisher, which was also rated highly by our respondents. Solomon (2014) similarly found that this factor was rated highly. Our proposal that OAMJs may gain a “reputational subsidy” from the publisher brand, and particularly from its high‐impact titles, seems to be supported by this (Spezi et al., 2017).

Linked to perceptions of quality, the JIF has often been seen as important in author choice of journal (Cope & Phillips, 2014) and our findings support that conclusion for all journal types, including OAMJs. Once again there were geographical differences: Chinese, Taiwanese, and Spanish authors gave the JIF higher priority than those from the United Kingdom, United States, or Canada, for example. This is likely to reflect national policy contexts in which authors are incentivized, often with financial rewards, to publish in high impact factor journals (Borrego & Anglada, 2016; Quan, Chen, & Shu, 2017; Shin, 2017). Our previous studies, which observed high proportions of articles authored by Chinese authors in *Scientific Reports* and *Medicine* (Wakeling et al., 2016, 2017), posited the importance of the impact factor to authors. Our findings here support that hypothesis. With regard to *Medicine*, we observed that the very high impact factor of the journal following its conversion to a mega‐journal model was in many respects a “hangover” from its previous highly‐selective approach, and therefore we expected its JIF to decline (which in fact has now taken place). A future study could usefully examine whether submissions to this journal from Chinese authors decline correspondingly.

The quality of the peer reviews produced for a journal was also highly valued by authors of all journal types. It was the second most important factor identified by OAMJ authors in selecting a journal behind the quality of the journal, and poor quality peer reviews appear to be the most likely thing to stop authors resubmitting. Significantly, this corroborates our findings from our academic focus groups (Wakeling et al., 2018). These discussions revealed that authors value peer review as a means to improving their articles and, linked to this, some were skeptical that soundness‐only peer review could provide such feedback. However, this valuing of peer review does not seem to be widely reflected in the literature on author motivations and experiences, and was not included as a factor in Solomon's survey (2014). Further work on the importance of this factor is required.

As well as the quality of peer review, the review criteria of the journal were also reported to be important to authors. This relates to RQ3, and our findings show that, paradoxically, as many as two‐thirds of OAMJ authors did not understand the criteria used for the review of their articles on submitting their article, believing that the journal had considered the significance/impact of their research. This is in many respects a startling finding. It is of course possible that some respondents misinterpreted the question, or might have been misled by recalling reviewer comments about their article that mentioned the significance or importance of their research. However, our view is that the question asked of survey respondents was clear (“When you submitted the article identified in the email invitation, were you aware which of the following peer review criteria were considered by this journal?”), and the available answer options for each peer review element were unambiguous (“Journal considers this,” “Journal does not consider this,” and “Don't know”). Thus, although there were potentially a small number of respondents who did not interpret the question as we intended (as is the case for any survey), we are confident that our results reflect a real and significant level of confusion among researchers about the peer review policies of OAMJs.

Although mega‐journal publishers have argued strongly for soundness‐only peer review and the issue has been hotly debated (Spezi et al., 2017), it is apparent that an understanding of the approach does not yet seem to have penetrated the scholarly community. This finding does, however, complement results from previous phases of our mega‐journals project. We found in our interviews with publishers and senior editors that reviewers (or even section editors) for OAMJs often took into account novelty, significance, and relevance of an article, rather than focusing on soundness only (Spezi et al., 2018). Based on these findings, some misunderstandings among authors might reasonably have been expected, but the levels of misunderstanding are remarkably high. Misunderstanding is highest in Taiwan, Brazil, India, and China, all of which are growing in importance in their contribution to global scholarly outputs; but even in the United States and United Kingdom, more than half of the authors evidently did not understand the OAMJ assessment criteria. This raises a number of interesting issues, such as the degree to which authors are properly researching the policies of journals before submission, and whether publishers, who typically communicate these peer‐review policies to authors on information pages of the journal website (for example, “About the journal,” Editorial Policies,” “Aims and Scope”), could display this information more prominently.

There is also a dilemma here for OAMJ publishers and advocates. Peer reviewers of OAMJ articles often include comment on novelty, significance, and relevance in their reviews, and although this is not what OAMJs require, authors seem to value highly the quality of peer review currently offered by OAMJs. If publishers pare down peer review reports to exclude judgments of factors other than soundness, there is a danger authors would view these reports less favorably, and their desire to publish in an OAMJ may diminish. There is then an apparent risk in OAMJ publishers attempting to limit peer review reports more strictly to soundness only unless they can ensure that authors clearly understand the value of the model—something that is not the case at present. The current response by OAMJ publishers to this problem of eliminating judgments of novelty, significance, and relevance from *acceptance* decisions, even if peer review reports include them, places a significant burden on academic editors to filter peer reviewer recommendations (perhaps even reversing recommendations to reject articles because of this), but may have some merit in maintaining author satisfaction over and above the pragmatic compromise it appears to be. However, it is notable that rather than “soundness‐only *peer review,*” this constitutes a system of “soundness‐only *acceptance.”*

One factor that may contribute to a lack of author understanding of OAMJ peer review criteria is cascade. In addressing RQ4 (“To what extent do authors target mega‐journals as publication venues of first choice?”), we found that 47.8% (±1.3%) of mega‐journal articles had previously been submitted elsewhere, a result close to the one reported by Solomon (52.6%; 2014). Although the survey results alone do not explain the rationale of authors who choose to submit articles rejected by traditional journals to mega‐journals, our other work (Wakeling et al., 2018) suggests that when faced with a rejection, authors typically look to resubmit to a journal of somewhat lower perceived quality or prestige. It seems likely that mega‐journals, many of which have JIFs that place them within the mid‐tier of journal rankings, are often selected as a result of this process. We have also identified that cascade may be important for some mega‐journals, and if articles are being cascaded from journals using conventional peer‐review criteria to an OAMJ using soundness‐only criteria, it would be less surprising that authors may not appreciate this. It is important to note, however, that cascade does not necessarily mean low quality. Cascade from a highly selective *Nature* title to *Scientific Reports* may still result in the latter publishing high‐quality articles. Although the proportion of cascade articles published in *Scientific Reports* (18.9%; ±2.1%) was lower than some other OAMJs, the journal's very large size means this equates to more than 3,000 articles per year. *Scientific Reports'* high JIF compared with other OAMJs (even those that can cascade from high‐JIF titles) is also likely to derive from other factors, particularly the reputation of the publishers, incentivizing both article submissions, and also citations.

OAMJs are, however, not a homogeneous group. *PeerJ* stands out in a number of ways in relation to all of our research questions. Authors value the fact it is OA, its low publication charge, and speed of publication comparatively highly; they value its JIF less. At the same time, *PeerJ* authors seem to have a better understanding of the peer‐review approach, with only 44.5% (±4.4%) believing the journal considers importance/significance among its criteria compared with 68.9% (±1.3%) across other OAMJs. It seems that a higher proportion of *PeerJ* authors understand and support the model it sustains. *PeerJ* authors also tend to be more satisfied with their experience (it has the highest percentage of authors, 92.5% [±2.5%], saying that they would be likely to submit another article there), although some other OAMJs also show very high rates in this area. Interestingly, although *PeerJ* has a wide scope covering all of life and health sciences, our previous bibliometric analysis showed that it has a disproportionate number of articles in the areas of ecology and computational biology (Wakeling et al., 2016). Although this may create a community around the journal, something our previous work showed to be crucial in author acceptance (Wakeling et al., 2018), it may at the same time have limited its growth. Also, as a new stand‐alone start‐up, it has not benefitted from any reputational subsidy from preexisting well‐regarded titles or publisher brand.

Other differences between OAMJ titles were apparent. With respect to OAMJ author satisfaction (RQ2), although overall levels of satisfaction of the experience of the production process were high across all titles, there was some variation. Some of this may relate to particular journal requirements, such as that of submitting data—an issue that merits further research. There were also relatively low levels of satisfaction for *PLoS One* and *Scientific Reports* in general, and in particular for speed of publication. Expectations in this area are likely to be high because OAMJs have often made speed of publication an explicit priority, but there is evidence from our previous work of challenges arising from the often rapid scaling of journals that put a strain on technical infrastructure, business processes, and human capacity (for example, recruiting reviewers and editors) (Wakeling, Spezi, Creaser, et al., 2017). This may go some way to explaining the slightly higher levels of negativity associated with the larger‐scale OAMJs.

The variation in responses we observed across different mega‐journals relating to all of our research questions supplements our earlier findings indicating that there is no such thing as a “typical” mega‐journal (Wakeling et al., 2016). Mega‐journals have different breadths of scope, geographic distributions of authors, levels of perceived prestige and reputation, citation distributions, motivations underpinning their launch, operating models, editorial structures, and methods of implementing soundness‐only peer review (Spezi et al., 2018; Wakeling et al., 2016, 2017; Wakeling, Spezi, Creaser, et al., 2017). To this list we can now add apparently quite different communities of authors, with variations in the factors motivating submission to the mega‐journal, and different levels of awareness of OAMJ characteristics. Although the term “open‐access mega‐journal” remains useful as a means of classifying a set of journals with broadly similar characteristics (particularly their approach to peer review), it does not describe a homogenous group. We suggest that further work towards understanding the differences and commonalities between titles and author responses to them is likely to offer further insight into the potential of this publishing model.

Despite such heterogeneity, our data show clearly that authors of OAMJ articles for the most part do not understand the mega‐journal publishing model, although this varies geographically and across different OAMJ titles. This is a surprising finding, but itself gives rise to another question: Does it matter? From one perspective, it might be argued that (at least some) OAMJs have proved to be successful regardless of author misunderstandings of the model, if success is judged in terms of levels of willingness of authors to contribute to the journals and levels of satisfaction with the experience. On the other hand, our previous research has demonstrated that many mega‐journal publishers in launching OAMJs were aiming to change the way scientific communication was done (Wakeling, Spezi, Creaser, et al., 2017; Wakeling, Spezi, Fry, et al., 2017). Soundness‐only peer review was a central part of this aim, as it is seen by many of its advocates as more objective and inclusive, and the shift of judgments of novelty, significance, and relevance downstream to be a “community” decision after publication, was seen as more “democratic” (Spezi et al., 2018). This, it was hoped, would contribute to a transition away from JIF‐driven publishing incentives that may encourage overemphasis on novelty and significance in reporting. In order for this ambitious aim to be realized, however, the informed participation of all of the actors—authors, reviewers, editors, and readers—is needed. That is clearly something not (yet) achieved.

## Conclusion

Although variations in responses associated with different mega‐journals reinforce the notion that such journals represent a heterogeneous group, nonetheless some broad conclusions can be drawn from our study. The increasing number and size of mega‐journal titles demonstrates that the first two of four criteria for defining OAMJs—large scale and broad subject coverage—have been accepted by subdivisions within the academic community, although concerns remain about their relationship with specific disciplinary communities. Similar conclusions apply, albeit with some geographical variations, to their OA nature. However, soundness‐only peer review—arguably the most distinctive and radical feature of OAMJs—is still widely misunderstood in the community. It will be interesting to see when, or even if, this situation will change.

## Supporting information


**Appendix 1:** Copy of the questionnaire

**Appendix 2:** Mega‐journals selected for the study

**Appendix 3:** Journals in sample, and distribution and response rates

**Appendix 4:** Factors influencing choice of journal

## Footnotes

1 An additional motivation for including *RSC Advances* in our sample was the fact that the journal transitioned from a subscription to an APC‐supported OA model in early 2017. The data we have gathered prior to this transition will provide a useful point of comparison for future studies of *RSC Advances* author views.

2
https://www.onlinesurveys.ac.uk/
