# Supplementary material for: Motivations, understandings, and experiences of open‐access mega‐journal authors: Results of a large‐scale survey
Source: J Assoc Inf Sci Technol. 2019 Jan 22;70(7):754–68. doi: 10.1002/asi.24154 (PMC6853193; doi:10.1002/asi.24154)
Supplement: Supplementary file 1 — Appendix 1: Copy of the questionnaire [file ASI-70-754-s001.docx]

# Appendix 1: Copy of the questionnaire

This research explores the factors influencing authors when choosing an academic journal for publication of their work. For more information please visit oamj.org.

The survey should take no more than 5 minutes of your time. We would like to know your views relating to the **article and journal indicated in your invitation email**.

All data will be treated confidentially, and no individuals will be identified in the analysis and reporting of the survey results. You may withdraw from the study at any time by closing the browser window without submitting your responses, or, after submission by contacting [lisu@lboro.ac.uk](mailto:lisu@lboro.ac.uk).

Please submit your response by **Friday 14 April 2017**.

If you have any questions about this survey please contact LISU (lisu@lboro.ac.uk).

1. **First of all we need your consent**

**Please tick here to confirm you agree to take part:**

**Now thinking about the paper specified in your invitation email, which of the subject areas below best describe(s) the disciplinary scope of the paper?** *Please select one or more subject areas. If you cannot find appropriate area(s), please click 'Other' and give details.*

**1.**

*a.* **Medical Sciences** *(select all that apply)*

Clinical medicine

Clinical dentistry

Anatomy & physiology

Nursing & paramedical studies

Health & community studies

Pharmacy & pharmacology

Other

If you selected Other, please specify:

*b.* **Life Sciences** *(select all that apply)*

Biosciences

Psychology & behavioural sciences

Earth, marine & environmental sciences

Veterinary science

Agriculture & forestry

Other

If you selected Other, please specify:

*c.* **Physical Sciences and Mathematics** *(select all that apply)*

Chemistry

Physics

Mathematics

General engineering

Chemical engineering

Mineral, metallurgy & materials engineering

Civil engineering

Electrical, electronic & computer engineering

Mechanical, aero & production engineering

Information technology & systems sciences & computer software engineering

Other

If you selected Other, please specify:

*d.* **Social Sciences, Humanities & Arts** *(select all that apply)*

Architecture, built environment & planning

Catering & hospitality management

Business & management studies

Economics

Geography

Social studies

Media studies

Humanities & language based studies

History

Archaeology

Modern languages

Design & creative arts

Education & Sports

Other

If you selected Other, please specify:

**Your choice of journal**

1. **Thinking about the article and journal identified in the email invitation, please rate the importance of the following factors relating to *quality and reputation* in your decision to submit this paper to this journal:**

Options for Q2-4: Not at all important, Slightly important, Moderately important, Very important, Extremely important, Don’t know/can’t remember, Not applicable.

Quality of the journal

Impact factor of the journal

Reputation of the publisher

Editor and / or editorial board

1. **Please rate the importance of the following *social and institutional factors* in your decision to submit this paper to this journal:**

External / institutional evaluation of your research

Recommendation of colleagues

Previous experience of this journal

1. **Please rate the importance of the following *journal features* in your decision to submit this paper to this journal:**

Speed of review and publication process

Review criteria of the journal

High quality peer reviews

The fact the journal is Open Access [OA titles only]

The fact the journal is not Open Access [non-OA titles only]

Cost of publication [OA titles only]

The fact that I can publish in this journal without charge [non-OA titles only]

Journal's policy regarding the publication of research data

1. **How would you describe the subject scope of this journal?**

Very broad

Broad

Specialised

Very specialised

Don't know

- 1. **How important was this scope in your decision to submit this paper to this journal?**

Not at all important

Slightly important

Moderately important

Very important

Extremely important

Don't know/can't

remember

Not applicable

1. **Had you previously submitted the same article elsewhere?**

Yes

No

- 1. **If yes, was (re)submission of the article to this journal made at the suggestion of the editor or publisher of the previous journal?**

Yes

No

Don’t know/can’t remember

**5**

1. **If you would like to add any comments about your decision to submit this paper to this journal, please use this space:**

**7.**

**Your experience of the publication process**

1. **Thinking about the article and journal identified in the email invitation, please rate each stage of the publication process of this paper**

**Options:** Very poor, Poor, Average/neutral, Good, Excellent, Don’t know/can’t remember, Not applicable

Pre-submission contact with the editor/editorial staff

Ease of online submission

Speed of peer review

Quality of peer review

Speed of acceptance following submission of revisions

Speed of publication following acceptance

Ease of preparing supplementary research data and material

Quality of the proofing and presentation of the article

Process for signing license agreement

Process for paying article processing or other charges [OA titles only]

**9a. Was a fee (APC) paid to make your article immediately open access? [non-OA titles only]**

Yes

No

Don’t know/can’t remember

Not applicable

**9b.** **What was the fee for publication (APC or article processing charge)? [OA titles only]**

Full cost (APC)

Partial cost (partial waiver of APC)

No cost (full waiver of APC)

No cost (Journal does not charge)

Don't know/can't remember

1. **If you would like to add any comments about your experience of the publication process, please use this space:**
2. **When you submitted the paper identified in the email invitation, were you aware which of the following peer review criteria were considered by this journal?**

Options: Journal considers this, Journal does not consider this, Don’t know

Novelty/originality of the research

Relevance/interest of the subject matter

Importance/significance of the research

Scientific/technical soundness of the research

Clarity of argument and expression

1. **How likely would you be to submit future manuscripts to the same journal?**

Very likely

Quite likely

Unsure

Not very likely

Not at all likely

**10.11.**

1. **How likely would you be to recommend this journal to colleagues?**

Very likely

Quite likely

Unsure

Not very likely

Not at all likely

1. **If you would like to comment on the reasons for your answers to Q12/13, please use this space:**

**Finally, please tell us about yourself**

1. **Which of the following best describes your institution/organisation?** *(please select one)*

University or college

Hospital or medical school

Research Institute

Government Industrial/commercial

Other

If you selected Other, please specify:

1. **In which country is your institution/organisation based? [free text]**
2. **For how long have you been conducting research?**

Fewer than 3 years

3-5 years

6-9 years

10-14 years

15-24 years

25 years or longer

Not applicable

1. **Approximately how many articles did you publish in 2016?17**

0

1-5

6-10

11-20

More than 20
