# Supplementary material for: Motivations, understandings, and experiences of open‐access mega‐journal authors: Results of a large‐scale survey
Source: J Assoc Inf Sci Technol. 2019 Jan 22;70(7):754–68. doi: 10.1002/asi.24154 (PMC6853193; doi:10.1002/asi.24154)
Supplement: Supplementary file 2 — Appendix 2: Mega‐journals selected for the study [file ASI-70-754-s002.docx]

# Appendix 2: Mega-journals selected for the study

|  |  |  |  |  | Output | |
| --- | --- | --- | --- | --- | --- | --- |
|  | Publisher | *Scopus* subject | 2015 SNIP | 2015 JIF | 2015 | 2016 |
| *AIP Advances* | American Institute of Physics | Physical Sciences | 0.586 | 1.444 | 939 | 1,268 |
| *Biology Open* | The Company of Biologists | Life Sciences | 1.243 | 2.135 | 182 | 209 |
| *BMC Research Notes* | BioMed Central | Health Sciences | 0.722 | n/a | 889 | 540 |
| *BMJ Open* | BMJ Publishing Group | Health Sciences | 1.202 | 2.562 | 1,427 | 1998 |
| *F1000 Research* | Faculty of 10000 | Life/Health Sciences | 0.262 | n/a | 279 | 684 |
| *FEBS Open Bio* | FEBS Press | Life Sciences | 0.612 | 2.101 | 111 | 106 |
| *Heliyon* | Elsevier | General | n/a | n/a | 29 | 156 |
| *Medicine* | Wolters Kluwer | Health Sciences | 1.024 | 2.133 | 1,989 | 3,275 |
| *PeerJ* | PeerJ | Health Sciences | 0.859 | 2.183 | 799 | 1,298 |
| *PLOS ONE* | Public Library of Science | Life/Health Sciences | 1.044 | 3.057 | 28,114 | 22,077 |
| *Royal Society Open Science* | The Royal Society | General | 0.822 | n/a | 246 | 416 |
| *SAGE Open* | SAGE | Social Sciences | 0.33 | n/a | 291 | 369 |
| *Sage Open Medicine* | SAGE | Health Sciences | n/a | n/a | 68 | 101 |
| *Scientific Reports* | Nature | General | 1.589 | 5.228 | 10,642 | 20,517 |
| *SpringerPlus* | Springer | General | 0.511 | 0.982 | 832 | 2,091 |
