# Supplementary material for: Motivations, understandings, and experiences of open‐access mega‐journal authors: Results of a large‐scale survey
Source: J Assoc Inf Sci Technol. 2019 Jan 22;70(7):754–68. doi: 10.1002/asi.24154 (PMC6853193; doi:10.1002/asi.24154)
Supplement: Supplementary file 3 — Appendix 3: Journals in sample, and distribution and response rates [file ASI-70-754-s003.docx]

# Appendix 3: Journals in sample, and distribution and response rates

| **Journal title** | | | **Broad subject** | **Manuscript Management System** | **2015 SNIP** | **Source of email addresses** | **No of email addresses captured** | **Emails sent** | **Failed delivery** | **Final responses** | |  |
| --- | --- | --- | --- | --- | --- | --- | --- | --- | --- | --- | --- | --- |
| **OAMJ** | | |  |  |  |  |  |  |  |  | |  |
| AIP Advances | | | Physical | PXP | 0.586 | J. website | 1,460 | 1,455 | 54 | 109 | |  |
| Biology Open | | | Life | BiO | 1.243 | PubMed | 352 | 352 | 11 | 71 | |  |
| BMC Research Notes | | | Health | Editorial Manager | 0.722 | PubMed | 6,649 | 6,649 | 507 | 436 | |  |
| BMJ Open | | | Health | ScholarOne | 1.202 | PubMed | 2,843 | 2,843 | 105 | 659 | |  |
| F1000 Research | | | Life/Health | F1000Research | 0.262 | PubMed | 814 | 814 | 34 | 160 | |  |
| FEBS Open Bio | | | Life | ScholarOne | 0.612 | PubMed | 203 | 203 | 4 | 27 | |  |
| Heliyon | | | General | Evise | n/a | PubMed | 199 | 199 | 4 | 37 | |  |
| Medicine | | | Health | Editorial Manager | 1.024 | PubMed | 3,055 | 3,055 | 60 | 227 | |  |
| PeerJ | | | Health | PeerJ | 0.859 | PubMed | 1,847 | 1,847 | 53 | 484 | |  |
| PLOS One* | | | Life/Health | PLOS | 1.044 | PubMed | 45,489 | 9,096 | 420 | 1,221 | |  |
| Royal Society Open Science | | | General | ScholarOne | 0.822 | PubMed | 602 | 602 | 25 | 148 | |  |
| SAGE Open | | | Social | ScholarOne | 0.33 | PubMed | 485 | 485 | 18 | 131 | |  |
| Sage Open Medicine | | | Health | ScholarOne | n/a | PubMed | 137 | 137 | 4 | 26 | |  |
| Scientific Reports* | | | General | eJournalPress | 1.589 | PubMed | 29,790 | 10,072 | 304 | 1,382 | |  |
| SpringerPlus* | | | General | Editorial Manager | 0.511 | PubMed | 12,529 | 9,828 | 501 | 633 | |  |
| **Broad scope - OA** | | |  |  |  |  |  |  |  |  | |  |
| eLife | | | Life/Health | eLife | 1.23 | PubMed | 2,331 | 2,331 | 57 | 570 | |  |
| Nature Communications | | | Life/Physical | eJournalPress | 2.922 | PubMed | 6,412 | 6,412 | 180 | 1,208 | |  |
| Science Advances | | | Health | Science | n/a | PubMed | 586 | 586 | 16 | 150 | |  |
| **Broad scope - subscription** | | |  |  |  |  |  |  |  |  | |  |
| RSC Advances* | | | Physical | ScholarOne | 0.812 | J. website | 20,476 | 9,999 | 295 | 1,089 | |  |
| **Open access** | | |  |  |  |  |  |  |  |  | |  |
| BioMed Research International | | | Life | Hindawi | 0.594 | PubMed | 4,716 | 4,716 | 131 | 483 | |  |
| BMC Musculoskeletal Disorders | | | Health | Editorial Manager | 1.074 | PubMed | 4,139 | 4,138 | 300 | 201 | |  |
| Demographic Research | | | Social | Max Planck Soc. | 1.069 | J. website | 464 | 464 | 26 | 132 | |  |
| IEEE Photonics Journal | | | Physical | ScholarOne | 1.07 | J. website | 220 | 220 | 6 | 21 | |  |
| Nanoscale Research Letters | | | Physical | Editorial Manager | 0.611 | PubMed | 3,314 | 3,313 | 103 | 123 | |  |
| Neural Regeneration Research | | | Life | Editorial Manager | 0.31 | PubMed | 743 | 743 | 29 | 94 | |  |
| Oncotarget | | | Health | eJournalPress | 1.071 | PubMed | 6,448 | 6,448 | 236 | 643 | |  |
| **Subscription** | | |  |  |  |  |  |  |  |  | |  |
| Antiquity | | | Social | ScholarOne | 1.018 | PubMed | 298 | 298 | 13 | 99 | |  |
| Applied Economics | | | Social | ScholarOne | 0.74 | J. website | 772 | 772 | 34 | 164 | |  |
| Continuum | | | Social | ScholarOne | 0.567 | PubMed | 129 | 129 | 6 | 35 | |  |
| Emerging Markets Finance and Trade | | | Social | ScholarOne | 0.716 | J. website | 321 | 321 | 8 | 32 | |  |
| European Planning Studies | | | Social | ScholarOne | 1.115 | PubMed | 154 | 154 | 17 | 51 | |  |
| Health Communication | | | Social | ScholarOne | 1.197 | PubMed | 147 | 147 | 1 | 41 | |  |
| International J. of Science Education | | | Social | ScholarOne | 1.192 | PubMed | 229 | 229 | 4 | 69 | |  |
| Journal of Health Psychology | | | Social | ScholarOne | 1.018 | PubMed | 183 | 183 | 7 | 45 | |  |
| Journal of Interpersonal Violence | | | Social | ScholarOne | 1.272 | PubMed | 236 | 235 | 5 | 58 | |  |
| Journal of Neurophysiology | | | Life | eJournalPress | 1.018 | PubMed | 443 | 443 | 14 | 56 | |  |
| Journal of Urban History | | | Social | ScholarOne | 1.107 | PubMed | 82 | 82 | 4 | 28 | |  |
| Journal of Virology | | | Life | eJournalPress | 1.114 | PubMed | 1,385 | 1,385 | 53 | 202 | |  |
| Philosophy of Science | | | Social | Editorial Manager | 1.259 | PubMed | 19 | 19 | 3 | 7 | |  |
| Physics of Plasmas | | | Physical | PXP | 0.612 | J. website | 1,039 | 1,039 | 33 | 163 | |  |
| Psychonomic Bulletin & Review | | | Social | ScholarOne | 1.243 | J. website | 302 | 302 | 17 | 54 | |  |
| The British Journal of Social Work | | | Social | ScholarOne | 1.307 | J. website | 96 | 96 | 5 | 30 | |  |
| The Journal of Chemical Physics | | | Physical | PXP | 0.727 | PubMed | 4 | 1 | 0 | 0 | |  |
| The Journal of Physical Chemistry B | | | Physical | ACS | 1.070 | PubMed | 100 | 100 | 1 | 16 | |  |
| Experimental & Therapeutic Medicine | | | Health | Spandidos | 0.603 | PubMed | 889 | 889 | 24 | 25 | |  |
| Vaccine | | | Health | Evise | 1.193 | PubMed | 723 | 723 | 63 | 127 | |  |
| Journal of Immunology | | | Health | eJournalPress | 1.223 | PubMed | 911 | 911 | 32 | 116 | |  |
|  |  | **Titles in original sample for which we were unable to harvest email addresses** | | | | | |  |  | |  | |
| Anthropologist | | | Social |  | 0.321 | - | - | - | - | - | |  |
| Bioorganic and Medicinal Chemistry | | | Health |  | 1.026 | - | - | - | - | - | |  |
| Economic Modelling | | | Social |  | 1.024 | - | - | - | - | - | |  |
| Epidemiology and Infection | | | Health |  | 1.024 | - | - | - | - | - | |  |
| Food Control | | | Life |  | 1.616 | - | - | - | - | - | |  |
| Health Expectations | | | Health |  | 1.095 | - | - | - | - | - | |  |
| Journal of Banking and Finance | | | Social |  | 1.588 | - | - | - | - | - | |  |
| Journal of Popular Culture | | | Social |  | 0.558 | - | - | - | - | - | |  |
| Small | | | Health |  | 1.592 | - | - | - | - | - | |  |
| **Total** | | |  |  |  |  | **164,765** | **95,465** | **3,827** | **11,883** | |  |

* Owing to the limitations of the mail merge software (MS Outlook) emails were limited to 10,000 messages per day. When this was discovered, a systematic random sample was taken from titles with over 10,000 email addresses.
