# Supplementary material for: Motivations, understandings, and experiences of open‐access mega‐journal authors: Results of a large‐scale survey
Source: J Assoc Inf Sci Technol. 2019 Jan 22;70(7):754–68. doi: 10.1002/asi.24154 (PMC6853193; doi:10.1002/asi.24154)
Supplement: Supplementary file 4 — Appendix 4: Factors influencing choice of journal [file ASI-70-754-s004.docx]

# Appendix 4: Factors influencing choice of journal

| Journal | n | Quality of the journal | High quality peer reviews | Review criteria of the journal | Speed of review & publication process | Repu-tation of the publi-sher | Impact factor of the journal | External / institutional evaluation of your research | Previous experience of this journal | The fact the journal is Open Access | Cost of public-ation | Policy re: publication of research data | Recomm-endation of colleagues | Editor and / or editorial board | Journal Scope |
| --- | --- | --- | --- | --- | --- | --- | --- | --- | --- | --- | --- | --- | --- | --- | --- |
| *AIP Advances* | 109 | 81% (±7%) | 82% (±7%) | 78% (±8%) | 77% (±8%) | 78% (±8%) | 61% (±9%) | 67% (±9%) | 59% (±9%) | 27% (±8%) | 39% (±9%) | 40% (±9%) | 41% (±9%) | 47% (±9%) | 41% (±9%) |
| *Biology Open* | 71 | 77% (±10%) | 75% (±10%) | 71% (±11%) | 69% (±11%) | 69% (±11%) | 33% (±11%) | 48% (±12%) | 42% (±11%) | 49% (±12%) | 36% (±11%) | 37% (±11%) | 30% (±11%) | 46% (±12%) | 30% (±11%) |
| *BMC Research Notes* | 436 | 77% (±4%) | 78% (±4%) | 74% (±4%) | 68% (±4%) | 71% (±4%) | 52% (±5%) | 62% (±5%) | 55% (±5%) | 68% (±4%) | 61% (±5%) | 56% (±5%) | 49% (±5%) | 46% (±5%) | 44% (±5%) |
| *BMJ Open* | 659 | 91% (±2%) | 78% (±3%) | 65% (±4%) | 69% (±4%) | 74% (±3%) | 65% (±4%) | 62% (±4%) | 58% (±4%) | 61% (±4%) | 40% (±4%) | 35% (±4%) | 47% (±4%) | 31% (±4%) | 42% (±4%) |
| *F1000 Research* | 160 | 64% (±7%) | 74% (±7%) | 68% (±7%) | 73% (±7%) | 57% (±8%) | 28% (±7%) | 52% (±8%) | 50% (±8%) | 67% (±7%) | 51% (±8%) | 52% (±8%) | 34% (±7%) | 44% (±8%) | 27% (±7%) |
| *FEBS Open Bio* | 27 | 89% (±12%) | 88% (±12%) | 77% (±16%) | 85% (±13%) | 70% (±17%) | 54% (±19%) | 65% (±18%) | 50% (±19%) | 44% (±19%) | 44% (±19%) | 54% (±19%) | 27% (±17%) | 41% (±19%) | 52% (±19%) |
| *Heliyon* | 37 | 80% (±13%) | 83% (±12%) | 72% (±14%) | 72% (±14%) | 72% (±14%) | 45% (±16%) | 56% (±16%) | 46% (±16%) | 47% (±16%) | 48% (±16%) | 61% (±16%) | 56% (±16%) | 47% (±16%) | 38% (±16%) |
| *Medicine* | 227 | 83% (±5%) | 79% (±5%) | 76% (±6%) | 80% (±5%) | 65% (±6%) | 83% (±5%) | 59% (±6%) | 66% (±6%) | 36% (±6%) | 47% (±6%) | 57% (±6%) | 39% (±6%) | 45% (±6%) | 43% (±6%) |
| *PeerJ* | 484 | 75% (±4%) | 80% (±4%) | 76% (±4%) | 80% (±4%) | 54% (±4%) | 40% (±4%) | 43% (±4%) | 60% (±4%) | 75% (±4%) | 70% (±4%) | 48% (±4%) | 42% (±4%) | 40% (±4%) | 23% (±4%) |
| *PLOS One* | 1,221 | 85% (±2%) | 79% (±2%) | 74% (±2%) | 74% (±2%) | 62% (±3%) | 66% (±3%) | 63% (±3%) | 60% (±3%) | 59% (±3%) | 40% (±3%) | 44% (±3%) | 43% (±3%) | 35% (±3%) | 29% (±3%) |
| *RS Open Science* | 148 | 80% (±6%) | 82% (±6%) | 78% (±7%) | 69% (±7%) | 76% (±7%) | 41% (±8%) | 43% (±8%) | 46% (±8%) | 60% (±8%) | 67% (±8%) | 45% (±8%) | 38% (±8%) | 39% (±8%) | 32% (±8%) |
| *SAGE Open* | 131 | 72% (±8%) | 79% (±7%) | 75% (±7%) | 75% (±7%) | 80% (±7%) | 50% (±9%) | 63% (±8%) | 56% (±9%) | 64% (±8%) | 59% (±8%) | 50% (±9%) | 43% (±8%) | 52% (±9%) | 40% (±8%) |
| *Sage Open Medicine* | 26 | 73% (±17%) | 85% (±14%) | 73% (±17%) | 65% (±18%) | 85% (±14%) | 48% (±19%) | 50% (±19%) | 44% (±19%) | 73% (±17%) | 60% (±19%) | 77% (±16%) | 30% (±18%) | 38% (±19%) | 42% (±19%) |
| *Scientific Reports* | 1,382 | 91% (±2%) | 84% (±2%) | 76% (±2%) | 71% (±2%) | 77% (±2%) | 78% (±2%) | 65% (±3%) | 57% (±3%) | 46% (±3%) | 36% (±3%) | 42% (±3%) | 43% (±3%) | 43% (±3%) | 38% (±3%) |
| *SpringerPlus* | 633 | 83% (±3%) | 82% (±3%) | 78% (±3%) | 80% (±3%) | 74% (±3%) | 66% (±4%) | 67% (±4%) | 56% (±4%) | 56% (±4%) | 59% (±4%) | 60% (±4%) | 48% (±4%) | 53% (±4%) | 46% (±4%) |
| **All OAMJs** | 5,751 | 84% (±1%) | 80% (±1%) | 74% (±1%) | 74% (±1%) | 70% (±1%) | 64% (±1%) | 61% (±1%) | 58% (±1%) | 57% (±1%) | 47% (±1%) | 46% (±1%) | 44% (±1%) | 41% (±1%) | 36% (±1%) |
